# Functional Anatomy of the Inferior Longitudinal Fasciculus: From Historical Reports to Current Hypotheses

**DOI:** 10.3389/fnana.2018.00077

**Published:** 2018-09-19

**Authors:** Guillaume Herbet, Ilyess Zemmoura, Hugues Duffau

**Affiliations:** ^1^Department of Neurosurgery, Gui de Chauliac Hospital, Montpellier University Medical Center, Montpellier, France; ^2^INSERM-1051, Team 4, Saint-Eloi Hospital, Institute for Neurosciences of Montpellier, Montpellier, France; ^3^University of Montpellier, Montpellier, France; ^4^Department of Neurosurgery, Tours University Medical Center, Tours, France; ^5^UMR 1253, iBrain, INSERM, University of Tours, Tours, France

**Keywords:** inferior longitudinal fasciculus, ventral pathway, visual agnosia, semantics, reading, emotion recognition, prosopagnosia, lexical retrieval

## Abstract

The inferior longitudinal fasciculus (ILF) is a long-range, associative white matter pathway that connects the occipital and temporal-occipital areas of the brain to the anterior temporal areas. In view of the ILF’s anatomic connections, it has been suggested that this pathway has a major role in a relatively large array of brain functions. Until recently, however, the literature data on these potential functions were scarce. Here, we review the key findings of recent anatomic, neuromodulation, and neuropsychological studies. We also summarize reports on how this tract is disrupted in a wide range of brain disorders, including psychopathologic, neurodevelopmental, and neurologic diseases. Our review reveals that the ILF is a multilayered, bidirectional tract involved in processing and modulating visual cues and thus in visually guided decisions and behaviors. Accordingly, sudden disruption of the ILF by neurologic insult is mainly associated with neuropsychological impairments of visual cognition (e.g., visual agnosia, prosopagnosia, and alexia). Furthermore, disruption of the ILF may constitute the pathophysiologic basis for visual hallucinations and socio-emotional impairments in schizophrenia, as well as emotional difficulties in autism spectrum disorder. Degeneration of the ILF in neurodegenerative diseases affecting the temporal lobe may explain (at least in part) the gradual onset of semantic and lexical access difficulties. Although some of the functions mediated by the ILF appear to be relatively lateralized, observations from neurosurgery suggest that disruption of the tract’s anterior portion can be dynamically compensated for by the contralateral portion. This might explain why bilateral disruption of the ILF in either acute or progressive disease is highly detrimental in neuropsychological terms.

## Introduction

Theinferior longitudinalfasciculus (ILF) is a long-range white matter pathway that primarily connects the occipital lobe of the brain with the anterior temporallobe (ATL). Although the first anatomic description of the ILF dates back to the beginning of the 19^th^ century (Reil, 1812; Burdach, 1822), scientific efforts to better delineate its connective properties (Catani et al., 2002, 2003; Mori et al., 2002; Wakana et al., 2007; Epelbaum et al., 2008; Gomez et al., 2015; Latini, 2015; Latini et al., 2017) and identify ILF-mediated brain functions have only recently been fruitful, thanks to parallel advances in diffusion tensor imaging (DTI) and anatomic dissection (Martino et al., 2010, 2013; Sarubbo et al., 2013). Until recently, the ILF’s functions were usually inferred by purely anatomic reasoning. For example, the ILF provides critical connections between occipital and anterior temporal areas, both of which are known to contribute to object recognition; despite the absence of substantive behavioral evidence, it has therefore been hypothesized that the ILF has a critical role in this process. This trend is now being reversed, as a broad range of scientific techniques and approaches are being used to study the healthy or damaged brain. However, the knowledge gained over the last decade has not been comprehensively reviewed. This knowledge is nevertheless absolutely vital for the refinement of anatomic-functional models of behavioral functions in which the ILF may be a critical pathway. Clearly identifying the role of the ILF should also facilitate our comprehension of psychopathologic, neurodevelopmental, and neurodegenerative conditions [e.g., schizophrenia, autism spectrum disorder (ASD), and semantic dementia (SD)] in which disruption of this tract can lead to cognitive disorders and abnormal behavior.

In the present review, we first summarize data on the topological organization of the ILF; these range from historical reports to the latest DTI and dissection studies that suggest a multilayered organization. We next focus on studies of both brain-damaged and neurologically healthy participants in which the ILF’s role in an array of visual-based functions has been highlighted. Alteration of the ILF’s connectivity leads to visual agnosia and prosopagnosia, semantic and lexical retrieval difficulties, alexia, and impairments in visual emotion recognition. We also review reports concerning the impact of disruption of the ILF in psychopathologic and other neurologic disorders; these data indicate how the tract may be involved in the pathogenesis of a range of clinical and behavioral symptoms. Lastly, we look at how the ILF (and especially its anterior part) can be dynamically compensated for in certain pathophysiologic situations.

## Review Criteria

Literature on the ILF published up to 2017 was accessed by searching open-access Internet and proprietary databases (PubMed, PubMed Central, MEDLINE, Web of Science, and Google Scholar). The search term was “inferior longitudinal fasciculus.” Records were screened manually, and only experimental investigations and reviews were selected. Historical reports not referenced in the databases were also selected for review.

## Anatomy

### Historical Descriptions

**FIGURE 1:**
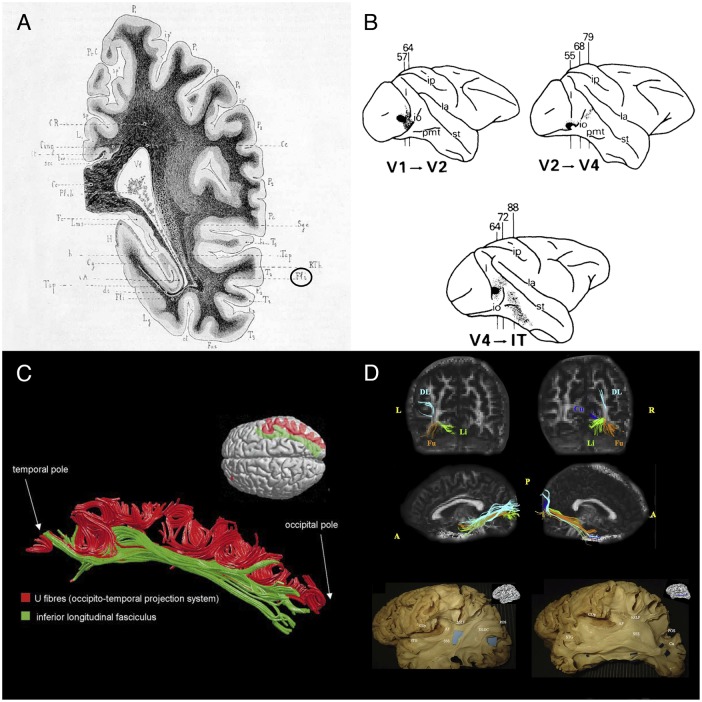
Historical and recent anatomic descriptions of the ILF. **(A)** A drawing of a coronal section passing through the ventricular atrium posterior to the caudate nucleus, showing the inferior longitudinal fasciculus (Fli) surrounding the optic radiations (RTh), the tapetum fibers (Tap), and the lateral ventricle (Vl). From Dejerine’s and Dejerine-Klumpke’s book “*Anatomiedes Centres Nerveux*,” 1895. Figure 383. **(B)** Reconstructions of autoradiographic observations. Injection sites and cortical projections in three monkeys are illustrated. Top left panel: injection (in black) into the striate cortex (V1), showing projection to V2 via U fibers and labeled fibers in the internal sagittal stratum that terminate subcortically in more rostral sections. Top right panel: injection into V2, showing projection to V4 via U fibers, labeled fibers in the internal sagittal stratum that terminate subcortically in more rostral sections, and labeled fibers in the tapetum that cross to the contralateral hemisphere. Bottom panel: injection into V4, showing projection to inferior temporal (IT) cortex via U fibers, labeled fibers in the internal sagittal stratum that terminate subcortically, and labeled fibers in the tapetum that cross to the contralateral hemisphere via the corpus callosum. Tusa and Ungerleider (1985) concluded that the ILF does not have long association fibers. **(C)** The ILF (in green) and the U-shaped fibers (in red) of the right hemisphere in a single brain dataset. The U-shaped fibers are located laterally to the ILF, and connect the adjacent gyri of the lateral occipitotemporal cortices to form the occipital-temporal projection system from Catani et al. (2003) **(D)** Subcomponents of the ILF. Top and middle rows: Diffusion tensor tractography of ILF subcomponents in an individual. Top row: a coronal view of the whole course of each segment (from the occipital terminations). Middle row: a sagittal view of a tractographic reconstruction of the four ILF subcomponents on the right side and only three segments on the left side. Bottom row: White matter dissection of the lateral aspect of a left hemisphere, showing the dorsolateral occipital segment (left panel) and the cuneal segment (right panel). Permission to reproduce the figures has been granted. A, anterior; AF, arcuate fasciculus; COp, central operculum; Cu, cuneal segment (blue); DL, dorsolateral occipital segment (turquoise); DLOC, dorsolateral occipital segment; Fu, fusiform segment (orange); hSLF, horizontal segment of the superior longitudinal fasciculus; L, left; Li, lingual segment (green); P, posterior; POS, parieto-occipital sulcus; R, right; SSS, sagittal stratum; STG, superior temporal gyrus.

Burdach (1819,1826) and Dejerine and Dejerine-Klumpke (1895) provided details of the tract’s structure in their important book “*Anatomie des centres nerveux*.” By this time, scientists had started to use dichromate to harden anatomic specimens, and the invention of the microtome had simplified the preparation of serial sections. The development of these and other histopathologic techniques (notably methods for staining myelin sheaths) and application of Wallerian degeneration had enabled the performance of remarkably accurate analyses long before the advent of MRI and diffusion tractography. Nonetheless, the Dejerines’ meticulously detailed description of the ILF was mostly restricted to the core of the tract, i.e., the compact portion of fibers surrounding the inferior and inferior-lateral aspects of the lateral ventricle. According to the Dejerines (**Figure 1A**), the ILF formed “a kind of sharply bent “gutter” or groove, open at the top and the inside, which receives fibers from the occipital-temporal lobe in its concave projection. […] Inside the occipital lobe, the inferior wall of the gutter turns in on itself at the top and on the inside. At the base of the *cuneus*, the two edges of the “gutter” are so close to each other that they join together and transform the bundle into a kind of empty cone, the slender apex of which is located around 2.5 cm from the occipital pole. It follows that on coronal sections, the ILF is shaped like a relatively irregular, angular ring inside the occipital lobe, and like a sharply bent “gutter” inside the temporal lobe.”

In view of the fibers’ cortical terminations and orientation, the Dejerines stated that the ILF “sends a great number of fibers to the three temporal convolutions (T1, T2, and T3), the third occipital convolution (O3), and the fusiform and lingual lobules.” The Dejerines further affirmed that “the fibers of the ILF originate inside the cortex of the occipital pole and lobe (…) and rapidly come together to form a thin annular fascicle behind the occipital horn (…), whereas fibers originating from the inferior-lateral half of the occipital lobe are oriented from back to front along the inferior-lateral edge of the occipital and temporal horns, fibers originating from the superior-lateral part of this lobe are oriented very obliquely downward and forward along the lateral wall of the ventricular atrium, and are all the more oblique when they come from higher regions.”

### Controversial Aspects

In their book, the Dejerines disagreed with the descriptions made by Gratiolet (1839), Meynert (1865), and Wernicke (1881), who considered that the ILF was made of projection fibers from the occipital lobe. In contrast, the Dejerines agreed with Sachs’ suggestion (in Sachs, 1992) whereby the ILF (referred to as the *stratum sagittale externum* by Sachs) was composed solely of association fibers. According to the Dejerines, the projection and association fibers in the superior part of the ILF are so intertwined that dissection cannot resolve the problem; the only way to distinguish between the two is to apply the Wallerian degeneration method. The Dejerines also stated that “the inferior portion of the ILF is solely an association pathway.” Nonetheless, the same question was raised a century later by Tusa and Ungerleider (1985); via blunt dissections of human and monkey brains, and autoradiography of monkey brain slices, the researchers only found a series of short U fibers connecting the occipital and temporal regions (**Figure 1B**). Tusa and Ungerleider (1985) therefore disagreed with Sachs’ and the Dejerines’ descriptions, although there is some neurophysiologic evidence (Wilson et al., 1983) of a direct connection between occipital and temporal lobe.

It was not until Catani et al.’s (2003) study using MR diffusion tractography (then a new method for investigating white matter tracts) that the hypothesis of a direct connection between the temporal and occipital lobes was again raised. These tractography experiments (**Figure 1C**) demonstrated that “in addition to the occipital-temporal projection system [as proposed by Tusa and Ungerleider (1985)], a major associative connection between the occipital lobe and ATL is provided by a fiber bundle whose origin, course, and termination are consistent with classical descriptions of the ILF.” This direct connection has been repeatedly identified in further studies using high-resolution DTI (e.g., Kamali et al., 2014; Duan et al., 2015; Keser et al., 2016).

### Does the ILF Have an Asymmetric, Multilayer Structure?

Subsequent studies of the ILF (Fernández-Miranda et al., 2008; De Benedictis et al., 2014; Latini, 2015; Latini et al., 2017) based on Klingler’s dissection method (a refinement of blunt dissection), providing reliable data on the anatomy of major fiber bundles (Zemmoura et al., 2016) and, in some cases, additional tractography (Latini et al., 2017; Panesar et al., 2018) not only confirmed the classical descriptions of the direct connection between occipital and temporal regions but also sought to detail the subcomponents of this association tract (**Figure 1D**). The most precise study was performed by Latini et al. (2017), who described marked lateralization of the ILF in the right hemisphere, and the presence of four consistently identified branches: a fusiform branch connecting the fusiform gyrus to the anterior temporal regions (from T1 to T5); a dorsolateral occipital branch connecting the superior, middle and inferior occipital gyri to the anterior temporal regions (from T2 to T4); a lingual branch connecting the lingual gyrus to the anterior part of the middle temporal gyrus (T2); and a minor cuneal branch connecting the cuneus to the anterior mesial temporal gyri T4 and T5.

### Anatomical Relationships of the ILF With Other White Matter Pathways

The ILF is in direct contact with five other association tracts: the uncinate fasciculus, the inferior fronto-occipital fasciculus (IFOF), the long and posterior/vertical segments of the arcuate fasciculus and the vertical occipital fasciculus of Wernicke. It has also close relationships with the optic radiations and with the fibers forming the tapetum of the corpus callosum.

Both the uncinate fasciculus and the ILF have cortical projections in anterior temporal structures. They provide an indirect anatomical connection between the posterior temporal and occipital areas, and the frontal lobe. According to some authors, both anatomical connectivities may form an indirect ventral stream for language and semantic processing (Duffau et al., 2013).

The IFOF is also in close relationship with the ILF. Inside the temporal and occipital lobes, the ILF runs more superficial and ventral than the IFOF. Both white matter tracts have cortical terminations in posterior occipito-temporal areas, especially in the fusiform, lingual and lateral occipital cortices.

The long and the posterior segments of the arcuate fasciculus, the vertical occipital fasciculus and the ILF have all four cortical terminations in the posterior part of the inferior temporal gyrus, making this brain area important for direct interactions between the ventral and dorsal streams.

Finally, at the deep aspects of the ILF, inside the temporal lobe, are found the fibers forming the *stratum sagittale* where pass the IFOF and the middle longitudinal fasciculus cranially, and the optic radiations and the tapetum medially.

### Anatomic Maturation

Diffusion tensor imaging studies of white matter maturation over the lifespan generally suggest that the ILF matures earlier than other association white matter fibers (e.g., the cingulum, the uncinate fasciculus, and the arcuate fasciculus) (Dubois et al., 2008; Lebel et al., 2008; Hasan et al., 2010; Lebel et al., 2012). In humans, the ILF can be identified at the age of three months on fractional anisotropy (FA) maps derived from DTI (Hermoye et al., 2006). Indeed, the ILF is the association tract that shows the earliest and fastest age-related changes in DTI parameters: the FA reaches 90% of its maximum by the age of 11 years, and the mean diffusivity (MD) falls to its minimum by the age of 20 (Lebel et al., 2008). The age-related increase in FA (a measure of tract integrity) and decrease in MD are thought to reflect white matter maturation processes (i.e., fiber organization, pre-myelination, and myelination; see Yoshida et al., 2013 and Dubois et al., 2014, for example). This could indicate that the early development of this tract is associated with the rapid maturation of some basic perceptual and cognitive processes (Lebel et al., 2012). These observations are in line with the ILF’s putative role in visual cognition (see below), aspects of which are present at birth. Indeed, visual cognition is a key part of early cognitive functioning, and develops rapidly during infancy (Rose et al., 2004). However, it is noteworthy that the ILF and other association tracts may mature later than originally thought. Indeed, the results of a recent longitudinal study showed that the ILF continues to develop during adolescence and is not fully mature until the age of 24 or so (Lebel and Beaulieu, 2011; Lebel and Deoni, 2018).

## Functions Mediated by the ILF

### Object Recognition

In view of the ILF’s cortical endpoints in visual occipital and inferior temporal associative areas, this tract is topographically well located to subserve the so-called ventral visual stream (as opposed to the dorsal visual stream) thought to be critical for object recognition (Ungerleider and Haxby, 1994; Milner and Goodale, 1995). In support of this hypothesis, unilateral or (more frequently) bilateral disruption of the ILF following infarction of the posterior cerebral artery (Benson et al., 1974; Albert et al., 1979; Feinberg et al., 1994) or occipital-temporal hematoma (Kawahata and Nagata, 1989; Meichtry et al., 2017) has been linked to visual agnosia (i.e., the inability to recognize objects, despite the absence of primary visual defects). These neuropsychological findings concur with the results of neuromodulation studies in which “virtual” disruption of the ILF (using direct electrostimulation) in patients undergoing “awake” craniotomy for low-grade glioma caused transient visual hemi-agnosia (Mandonnet et al., 2009; Coello et al., 2013; Gil-Robles et al., 2013). In patients with posterior cortical atrophy (a rare neurodegenerative condition characterized by progressive focal degeneration of the posterior cortex), one observes (i) a decrease in cortical volume in the occipito-temporal areas, and (ii) structural disruption of the ILF (as evidenced by significant increases in mean, parallel and transverse diffusivities) (Migliaccio et al., 2012). As a consequence, progressive degeneration of the ILF may well be involved in the pathogenesis of the high-level visual impairments typically observed in these patients, and especially visual agnosia (Benson et al., 1988). In a more indirect argument, children with a developmentally impaired ability to recognize objects also show bilateral disruption in the ILF (as evidenced by low FA compared to age-matched controls) - the extent of which is correlated with the severity of the impairment (Ortibus et al., 2012). Taken as a whole, these findings clearly indicate that (i) the ILF carries signals that are important for object recognition, and (ii) disruption of this pathway can lead to visual agnosia - especially when the disruption extends to both hemispheres.

### Face Recognition

Converging evidence from both positron emission tomography and functional MRI (fMRI) studies suggests that certain cortical regions in the ventral occipital-temporal cortex are part of a “core” face-selective network, i.e., they are more strongly activated by faces than by other classes of stimuli like objects or places (for a comprehensive review, see Grill-Spector et al., 2017). The first of these regions is located in the inferior occipital gyrus (the occipital face area, OFA), whereas the two others are situated more anteriorly in the posterior and middle parts of the fusiform gyrus (the fusiform face area, FFA). Although other regions (especially the ATL) are reportedly face-selective, they are currently considered to belong to the so-called “extended” face network.

Given the cortical anatomies of the “core” and “extended” face networks, it is likely that the ILF and some of the shorter fibers in the occipital-temporal projection system (Tusa and Ungerleider, 1985; Catani et al., 2003) convey critical signals in face perception and recognition. This may especially be the case in the right hemisphere, which is known to have a prominent role in face processing. In fact, the results of two DTI studies of healthy subjects suggest that the ILF indeed carries critical signals for face processing. In the first study, face recognition performance was strongly associated with higher fractional anisotropy (FA) in the anterior part of the right ILF, whereas scene recognition performance tended to be correlated with the FA in the middle and posterior parts of both the left and right ILFs (Tavor et al., 2014). According to the second DTI study, there is a double dissociation between the functions of the right ILF and those of the fornix. More specifically, DTI parameters (including MD and FA) in the ILF (but not in the fornix) were strongly associated with face processing, whereas the same parameters in the fornix (but not in the ILF) were correlated with scene processing (Hodgetts et al., 2015).

If the ILF does indeed have a significant role in face processing, then disruption of this pathway should be associated with impaired face recognition. The best evidence of this relationship has come from studies of patients with prosopagnosia, i.e., in whom face recognition is severely impaired despite normal vision and intelligence. A recent study found that patients with congenital prosopagnosia displayed a notable reduction (relative to healthy subjects) in the microstructural organization of both the ILF and the IFOF on both body sides (Thomas et al., 2009). Importantly, the mean FA values in the right ILF and (to a lesser extent) the right IFOF were inversely correlated with impaired face recognition. In a further study, a patient suffering from progressive prosopagnosia showed fewer streamlines in the right ILF (but not the left ILF or the IFOF) than matched control subjects did (Grossi et al., 2014). However, two other studies found that white matter disruption in the vicinity of the middle FFA (rather than disruption of the entire tract) may be a better predictor of face recognition impairments in individuals with prosopagnosia (Gomez et al., 2015; Song et al., 2015). Lastly, a study revealed that the accuracy of face memory was predicted by the microstructural properties of the right ILF; more specifically, lower FA was associated with better performance (Unger et al., 2016).

In summary, the result studies of healthy individuals and brain-damaged patients suggest strongly that a functional right ILF is crucial for efficient face recognition. However, this hypothesis does not rule out the possible involvement of other white matter connectivities (whether associative or intralobar) in face recognition. Indeed, functional imaging has identified a number of face-selective cortical areas. For example, white matter connections between the right posterior superior temporal sulcus and the temporal pole (both of which belong to the “extended” face network) may convey critical signals for face recognition–perhaps through the fibers in the middle longitudinal fasciculus.

### Reading

Regions in the ventral occipital-temporal cortex have been repeatedly identified as being particularly important in reading processing; this is especially true for the so-called visual word form area (vWFA) located in the lateral occipitotemporal sulcus (between fusiform and inferior temporal gyri), which enables the recognition of word forms (Dehaene et al., 2002; Dehaene and Cohen, 2011). The fact that lesions in this area are known to cause alexia (Hillis et al., 2005; Tsapkini and Rapp, 2010; Turkeltaub et al., 2014) demonstrates the region’s causal involvement in reading. Although the anatomic connectivity of the vWFA has not yet been well established (Yeatman et al., 2014), there is some evidence to suggest that the posterior part of the left ILF conveys critical, reading-related visual information from occipital areas to the posterior vWFA. In one study, progressive degeneration of the ILF was observed in a patient who developed pure alexia after surgical resection posterior to the vWFA–suggesting that differentiation of the posterior vWFA is the pathophysiologic cause of reading difficulties (Gaillard et al., 2006; Epelbaum et al., 2008). This hypothesis was supported by a recent case report on the advent of pure alexia following surgery-related disruption of the left posterior ILF (Zemmoura et al., 2016).

In line with the hypothesis whereby the ILF subserves the semantic/orthographic reading pathway, other data suggest that the tract is involved in reading comprehension. For example, inter-individual variations in the microstructural properties of both the left and right ILFs in people with normal intelligence are correlated with word reading fluency and reading comprehension (Horowitz-Kraus et al., 2014). This agrees with the fact that difficulties in reading comprehension were found to be associated with abnormalities of the right ILF in adolescents born preterm (Feldman et al., 2012) and abnormalities of the left ILF in poor decoders (Arrington et al., 2017). Lastly, these findings are consistent with the observation that the rate of maturation of the ILF covaries with children’s reading abilities (Yeatman et al., 2012).

### Lexical and Semantic Processes

The ILF has many connections to the ATL; it has been suggested that the latter’s ventrolateral aspect ATL acts as a transmodal semantic hub (Visser et al., 2010; Binney et al., 2012; Lambon Ralph, 2014; Rice et al., 2015; Lambon Ralph et al., 2017). In view of this anatomic background, it has been suggested that along with other ventral white matter tracts [especially the IFOF and the uncinate fasciculus (UF)], the ILF acts as a major white matter pathway for the so-called semantic ventral stream (Saur et al., 2008; Turken and Dronkers, 2011; Duffau et al., 2013; Moritz-Gasser et al., 2013; Dick et al., 2014; Almairac et al., 2015). However, the ILF’s exact role in semantic cognition has not been yet firmly established. In fact, most of the evidence comes from patients with SD (Agosta et al., 2010; Acosta-Cabronero et al., 2011) or the semantic variant of primary progressive aphasia (svPPA), in whom the ILF is disrupted (Mandelli et al., 2014; D’Anna et al., 2016; Tu et al., 2016). Progressive degeneration of the ILF may account (at least in part) for the semantic impairments typically observed in these patients. However, to the best of our knowledge, only a few studies have revealed clear associations between changes in DTI parameters in the ILF and impaired semantic or lexicosemantic abilities. For example, a studies of patients with PPA highlighted significant positive correlations between performance in behavioral tasks probing semantic association abilities and word comprehension on one hand, and FA in the anterior left ILF on the other (Mandelli et al., 2014). In another study, significant correlations were found between some DTI metrics in the ILF [especially FA and radial diffusivity (RD)] and two semantic factors (semantic richness in the left ILF only, and semantic features in the left and right ILFs). This finding suggests that some of the lexicosemantic impairments observed in these patients may be partly explained by structural disruption of the ILF (Marcotte et al., 2017). Similar observations were made in patients with SD; MD in the left ILF was negatively correlated with worse performance in a lexicosemantic task (Agosta et al., 2010).

Two very recent studies of healthy individuals have provided a better understanding of the types of semantic processes that may be distributed through the ILF. In the first study, inter-individual variability in the microstructural properties of both the ILF and the UF was found to be predictive of successful word-learning; the significant negative correlation between performance and RD suggested that connectivities of the ILF and the UF are involved in the acquisition of novel word-to-meaning mappings, and thus in semantic learning (Ripollés et al., 2017). In the second study, the combined use of constrained spherical deconvolution-based tractography and behavioral assessments revealed a double correlational dissociation: inter-individual variability in the microstructural properties of the ILF was correlated with the richness of semantic autobiographical memory (i.e., a negative correlation between MD and performance), whereas inter-individual variability in the microstructural properties of the fornix was correlated with the richness of episodic memory (i.e., a positive correlation between FA and performance) (Hodgetts et al., 2017). Hodgetts et al. (2017) pointed out that this double dissociation is reminiscent of observations in patients with SD; although their semantic autobiographical memory is impaired, their episodic memory is relatively unaffected.

Other experimental evidence suggests that the left ILF is involved in other language processes–lexical retrieval, in particular. In a large-scale neuropsychological study of 110 patients having undergone a surgical resection for diffuse low-grade glioma (DLGG), residual tumor infiltration in the left ILF was found to be a strong predictor of permanent impairments in lexical retrieval (Herbet et al., 2016b). This result fits well with the observation whereby disruption of the left ILF in stroke patients (estimated as the likelihood of fiber tract disconnection) was predictive of naming impairments for certain non-unique categories (i.e., animals, fruits and vegetables) (Mehta et al., 2016). More anecdotally, a recent neurosurgical case study found that tumor invasion of the left ILF was associated with naming impairments (Shinoura et al., 2010).

### Emotions and Related Processes

By virtue of the ILF’s connections with both the visual cortex and the amygdala, this tract is conceptually well suited to the integration of visual and emotional processes within the cortical affective network. In fact, the first evidence for this involvement was reported in the 1980s and 1990s; three patients with occipital-temporal lesions extending to the ILF were described as suffering from a syndrome called visual hypoemotionality, defined as a “modality-specific inability to become aroused by visual cues” and which was accompanied by feelings of “unreality or detachment” (Bauer, 1982; Habib, 1986; Lopera and Ardila, 1992; Sierra and Berrios, 1998; Ross, 2008; Perez et al., 2014). At the time, this neuropsychological disturbance was interpreted as a “visuolimbic disconnection syndrome.” Recently, a case report on a fourth patient with a right parietal–temporal–occipital lesion has been published. Disruption of the patient’s right ILF was found to be significantly associated with low numbers and volumes of streamlines (compared with matched neurologically healthy controls). This finding adds support to the concept in which disruption of the amygdalo-occipital pathway may be the pathophysiologic basis of visual hypoemotionality (Fischer et al., 2016).

Other lines of inquiry suggest that the ILF and other ventral tracts may be particularly important for the recognition of facial emotions. The most robust findings in this respect come from two large-scale neuropsychological studies. In the first study (*n* = 104 patients), the extent to which the IFOF and/or the ILF were disrupted (with regard to lesion volume) was found to be strongly predictive of inability to identity facial emotions (Philippi et al., 2009). These results were confirmed in a second study of patients (*n* = 42) suffering from moderate-to-severe traumatic brain injury (Genova et al., 2015). Indeed, impaired performance in the Facial Emotion Identification Test was significantly and negatively correlated with several DTI parameters (notably axial diffusivity (AD), MD, and RD). On the same lines, a study of patients with amyotrophic lateral sclerosis found that difficulties in recognizing negative emotions were linked to microstructural changes (as evidenced by low FA) in both the right ILF and IFOF (Crespi et al., 2014). These studies’ general findings are in agreement with a recent report showing that the microstructure of both tracts predicts the accuracy with which facial emotions are discriminated (i.e., a negative correlation between performance and AD) (Unger et al., 2016). This also agrees with the fact that individuals who discriminate facial emotions (especially angry faces) more rapidly are those with a higher degree of macrostructural organization in the right ILF (Marstaller et al., 2016). Lastly, a study of people with antisocial personality disorder (a psychologic condition in which emotion regulation and facial recognition may be impaired), the microstructural properties of the left ILF (as measured by the RD) and two other white matter tracts were strongly correlated with risk behavior (Jiang et al., 2017).

### Visual Memory

A study of a 47-year-old patient with a tumor that had mainly invading the white matter of the temporal lobe suggested that the ILF may be involved in visual memory (Shinoura et al., 2007). The patient showed poor visual memory, as assessed by the revised version of the Wechsler Memory Scale. DTI evidenced the partial disconnection of the right ILF. This finding fits well with the proposed role of the ILF in relaying visually learned information during haptic processing (Lee Masson et al., 2017a) and in visual-haptic processing more generally (Lee Masson et al., 2017b).

### Summary

Taken as a whole, the studies reviewed here indicate that the ILF may be involved a broad range of brain functions concerning the visual modality, including object, face and place processing, reading, lexical and semantic processing, emotion processing, and visual memory. The data suggest that the ILF is a multi-functional white matter pathway that is mainly involved in visually guided behavior. Although some of the above-mentioned processes are markedly biased toward a particular hemisphere (e.g., the right ILF in face processing and left ILF in lexical processing and reading), other appears to be bilateral (e.g., object recognition). Further research must now better delineate the ILF’s exact state of involvement in each hemisphere.

It is also noteworthy that only a few studies have focused on the ILF’s functions. Much of the experimental evidence has been derived by studying the correlation between DTI scalars and behavior in healthy subjects. Accordingly, causal and converging evidence is still needed to confirm and generalize most of the results described in this section.

## Disruption of the ILF in Schizophrenia, Asd, and Other Neurologic Conditions

### Schizophrenia and Related Psychopathologic Disorders

Several lines of evidence suggest that white matter abnormalities are central in the pathogenesis of schizophrenia, and may result from impaired myelination and/or oligodendroglial function (Davis et al., 2003). Although these changes are usually described as (i) being widespread in both early- and late-onset forms of schizophrenia (Douaud et al., 2007; Price et al., 2007; Kanaan et al., 2009; Lee et al., 2013; Kelly et al., 2017) and (ii) concerning all types of white matter connections, recent meta-analyses suggest that certain white matter tracts are particularly compromised. This is especially the case for white matter tracts forming part of the frontotemporal connectivity (i.e., the UF, arcuate fasciculus, cingulum, and IFOF) or inter-hemispheric connectivity (the corpus callosum and anterior commissure) (Kubicki et al., 2005; Kelly et al., 2017). Some studies have linked these patterns of white matter disruptions to the neuropsychological impairments typically observed in patients with schizophrenia (impaired psychomotor speed, working memory, and executive functions, among others) (Lee et al., 2013; Kochunov et al., 2017).

Although structural disruption of the ILF is apparently less frequent than structural disruption of other white matter tracts, some studies suggest that ILF is only damaged in patients showing specific patterns of clinical symptoms (Ashtari et al., 2007; Cheung et al., 2008; Friedman et al., 2008; Phillips et al., 2009). This is consistent with the fact that patients with schizophrenia do not generally suffer from abnormal cognitive processes potentially related to alterations in the neural systems mediated by the ILF.

In a recent study, the mean DTI metrics (especially the FA) in the left ILF (but not the right ILF) were significantly lower in adolescents with schizophrenia than in healthy controls (Ashtari et al., 2007). Importantly, the investigators compared two subgroups of patients with or without a history of visual hallucinations. Ashtari et al. found that the mean FA in the left ILF was lower in the subgroup with a history of visual hallucinations than in the subgroup without history of visual hallucinations - suggesting that disruption of ILF may be a neuropathologic basis for visual hallucinations. A second study of 23 patients confirmed the structural disruption of ILF (on both the left and right), and highlighted a negative correlation between the mean FA in the right ILF and the severity of schizophrenic symptoms (Phillips et al., 2009). This finding was recently confirmed in a study of patients with early-course schizophrenia (Seitz et al., 2016).

Along the same lines, patients with 22q11.2 deletion syndrome (a genetic neurodevelopmental disorder associated with a range of physical, physiologic, neuropsychological, and psychiatric impairments) also display abnormal white matter connectivity. Importantly, individuals with 22q11.2 deletion syndrome have an elevated risk of developing schizophrenia, with an incidence of approximately 30%. Overall, individuals with 22q11.2 deletion syndrome appear to have the same patterns of white matter alterations as patients with schizophrenia (Dennis and Thompson, 2013; Scariati et al., 2016; Olszewski et al., 2017; Padula et al., 2017). Interestingly, ILF abnormalities have been consistently identified, and a recent study based on DTI and machine-learning classification (a support vector machine) not only confirmed that the ILF is one of the most affected tracts in 22q11.2 syndrome but also showed that the ILF’s DTI parameters are correlated with prodromal symptoms of psychosis; this suggests that disruption of the tract may increase the likelihood of developing schizophrenia-like disorders (Tylee et al., 2017). Among the broad array of neuropsychological disorders typically present in patients with 22q11.2 deletion syndrome, impairments of social cognition (including theory of mind and empathy) are rather common (Weinberger et al., 2016; Norkett et al., 2017; Olszewski et al., 2017), and were recently described as a powerful predictor of psychotic symptoms in this population (Jalbrzikowski et al., 2014). Given that the ILF is known to connect regions required for social cognition (such as the amygdala and fusiform gyrus), it is possible that abnormal DTI parameters in the ILF reflect a pathophysiologic mechanism accounting for (at least in part) social cognition disturbances and psychotic symptoms in this specific patient population. However, this hypothesis has yet to be specifically tested.

### Autism Spectrum Disorder

Disruption of the structural connectome is one of the most frequently reported features of ASD. Indeed, scientific research has repeatedly identified anomalies in the microstructural organization of associative and inter-hemispheric white matter tracts in this patient population. Indeed, fiber tract disruption is now considered to be an integral part of the neural phenotype in ASD. However, the hypothesis whereby patients with ASD present with widespread, severe alterations of white matter pathways has been challenged. For example, recent work demonstrated that most of the white matter changes observed in ASD might be artifacts caused by the patient’s head motion–a variable that had not been carefully controlled for in most of the literature studies (Koldewyn et al., 2014). However, Koldewyn et al.’s (2014) study also showed that the right ILF was the only tract found unambiguously to be damaged after controlling for the imaging quality. From a clinical perspective, this general finding fits very well with the fact that the right ILF is known to be a critical white matter tract for face and emotion processing (see above) and that impaired face and emotion recognition are neuropsychological markers of patients with ASD (Dawson et al., 2004; Weigelt et al., 2012). The selective disruption of the right ILF in ASD was recently confirmed (Boets et al., 2018). Interestingly, disruption to the ILF (as evidenced by lower FA) was correlated with slower performance in a visual search task. This finding is consistent with the ILF’s known role in visual cognition. Lastly, it is noteworthy that autistic patients with poor language skills display lower FA in the posterior part of the left ILF than autistic patients with moderate to strong language skills (Naigles et al., 2017), and that autistic traits in the non-clinical population correlate with the microstructure of the left ILF (i.e., FA values were positively correlated with the autism-spectrum quotient) (Bradstreet et al., 2017).

### Summary

In the above section, we reviewed evidence showing that disruption of the ILF is associated with specific patterns of neuropsychological or clinical symptoms in certain patient populations. In particular, ILF dysfunction may be involved in the pathogenesis of psychotic symptoms in both schizophrenia and 22q11.2 deletion syndrome, and may have a role in the occurrence of the social cognition impairments that characterize both of these disorders. However, the link between ILF dysfunction, behavioral outcomes, and social cognition impairments remains unclear and must be specifically addressed in future studies; ideally, a comprehensive battery of neuropsychological tests should be used to assess the different aspects of social cognition.

In accordance with the ILF’s putative functions, our review of the literature suggested that disruption of this tract in ASD can be associated with impaired visual perception and/or poor language skills. Nonetheless, the correlational evidence is relatively dispersed and must be confirmed in subsequent research. Our review raises a number of other interesting ideas, however. For example, it would especially useful to assess to what extent the impairments in face identification and emotion recognition observed in ASD are attributable to anomalies in the ILF–especially in the right hemisphere.

Lastly, the evidence reviewed here suggests that epilepsy-related dysfunction of the ILF may be a pathophysiologic correlate of a number of cognitive impairments in patients with TLE. However, the data are scarce, and little is presently known about the neuropsychological consequences of disruption of the ILF in this form of epilepsy. A more comprehensive assessment of this putative link might tell us more about the ILF’s functions in normal (non-clinical) circumstances.

## Plasticity of the ILF

As DLGG frequently affects structures in the ATL and thus the underlying connectivity (Duffau and Capelle, 2004; Herbet et al., 2016a), the anterior part of the ILF is often targeted for resection during “awake surgery” with stimulation mapping. However, axonal stimulation studies have failed to identify a role of this pathway in verbal language and semantics–two cognitive domains that are usually mapped and monitored in the operating theater (Mandonnet et al., 2007). Although these negative results should not be construed as evidence that the ILF is not (or only slightly) involved in these functions, they do suggest that ILF functions may be dynamically compensated for by other structures –especially since DLGG is known to induce an efficient neuroplastic response by the brain (Duffau, 2005; Mandonnet et al., 2007; Herbet et al., 2016b). This kind of interpretation may explained why patients with unilateral damage to the ATL (e.g., following a vascular stroke or surgical resection for a tumor or epilepsy) show only mild to moderate impairments in semantic memory (Lambon Ralph et al., 2010), even when the ATL is thought to be critical for this function. It is most likely that both the left and right ATLs have critical roles in semantic memory, and that this massive bilateral organization makes the semantic system more resistant to damage (Lambon Ralph et al., 2010; Rice et al., 2015). Indeed, the contralateral (undamaged) ATL may functionally compensate for the damaged ATL. In the event of bilateral damage to the ATL (as seen in neurodegenerative conditions), marked semantic disorders may appear. The same reasoning might apply to the ILF, which has dense cortical terminations in the ATL.

In this respect, a recent case study of a patient suffering from multifocal DLGG has provided a better understanding of potential plasticity of the ILF (Corrivetti et al., 2017). The patient underwent two operations. The first operation concerned the right occipital-temporal cortex, including the fusiform and inferior occipital gyri, and the ILF. The second operation (performed one year later) concerned the left anterior temporal-insular cortex, including the anterior ILF. Despite surgical resection of the OFA, the FFA and the ILF, the first operation did not lead to prosopagnosia. In contrast, the second operation caused severe, permanent prosopagnosia. These observations suggest that the face perception network (which is classically biased toward the right hemisphere) was reorganized within the left hemisphere, leading to prosopagnosia only when the left ILF was resected.

Along the same lines, a recent study showed that axonal stimulation of the anterior-to-middle part of the left ILF systematically induced lexical retrieval difficulties during a picture-naming task (i.e., anomia) but only when the temporal pole was not damaged by the tumor (i.e., when the pole was damaged, no impairments were evoked) (Herbet et al., 2018). As well as having confirmed that the left ILF is involved in lexical access, this finding suggests that the information broadcasted by this tract can be rerouted along alternative pathways when progressive tumor infiltration of the temporal pole prompts the latter to abandon its function via progressive functional compensation.

Although disruption of ILF may be compensated for under certain pathophysiologic circumstances, it may also serve as the basis for functional recovery in the event of partial damage. In a kurtosis-based tractography study, it was shown that intensive language rehabilitation (based on confrontation naming) reinforced the microstructural organization of the left ILF. Furthermore, this language rehabilitation was associated with a significant reduction in the naming error rate (i.e., a negative correlation between mean kurtosis and the frequency of semantic paraphasia), suggesting that therapy-induced structural plasticity of the left ILF might be the physiologic basis of semantic recovery in stroke patients (McKinnon et al., 2017).

Lastly, a recent study in neurologically intact participants showed that learning Morse code improves the microstructural organization of the left ILF and suggested that the acquisition of a new language skill is underpinned by the neuroplastic potential of this ventral tract (Schlaffke et al., 2017). In the latter study, the mean FA in the left ILF increased significantly between the pre-and post-learning periods, and the increase was correlated with behavioral performance.

## Futures Directions and Conclusion

**FIGURE 2:**
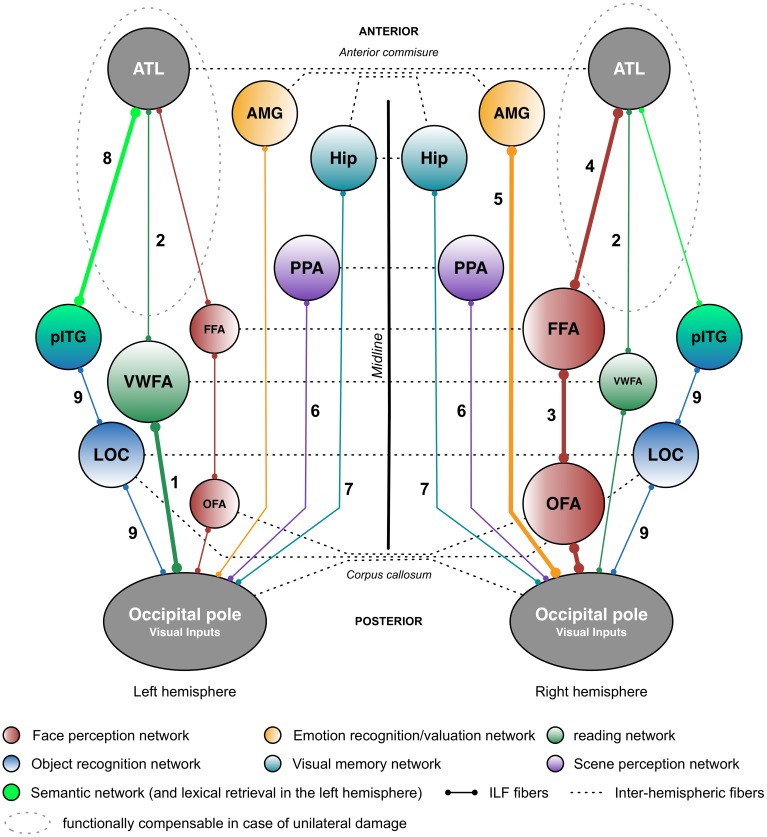
Putative functional pathways supported by the short and long fibers of the ILF. This schematic summary is derived from the recent literature data (mainly neuropsychological studies) on the ILF’s functions. Functional pathways that are biased toward a particular hemisphere are represented by thicker lines and larger circles. The neuropsychological syndromes in the event of disruption are as follows: (1) pure alexia; (2) reading comprehension; (3) apperceptive prosopagnosia; (4) associative prosopagnosia; (5) impairments in emotion recognition or hypoemotionality; (6) scene perception impairments; (7) visual memory impairments; (8) semantic and/or lexical retrieval impairments; and (9) visual agnosia. AMG: amygdala; ATL: anterior temporal lobe; FFA: fusiform face area; Hip: hippocampus; LOC, lateral occipital cortex; OFA: occipital face area; pITG: posterior inferior temporal gyrus; PPA, parahippocampal place area; vWFA, visual word form area.

Although the investigation of the ILF is still in its infancy, the available literature data nevertheless suggests that this white matter tract mainly supports visual functions; indeed, disruption of the ILF is associated with an array of neuropsychological syndromes that involve the visual modality. The findings reviewed here also suggest that the ILF has a multilayered functional organization (see **Figure 2** for a schematic illustration), which fits well with the tract’s many known or supposed cortical connections. Although some functions of the ILF appear to be relatively lateralized (e.g., reading in the left hemisphere, and face perception in the right hemisphere), a few reports suggest that the anterior part can be dynamically compensated for by the contralateral part. This would explain why bilateral disruption of the ILF appears to be an important feature in the emergence of severe neuropsychological impairments, as is notably the case in neurodegenerative diseases (e.g., SD and PPA) that typically affect the two hemispheres (albeit asymmetrically). Abnormalities in the ILF appear also to be an important determinant of cognitive and behavioral disturbances in certain psychopathologic or neurodevelopmental disorders, such as ASD or schizophrenia.

In general, one must bear in mind that few studies have directly investigated the relationships between the ILF’s microstructural properties and behavioral functions in both healthy and brain-damaged patients. Hence, most of the findings reported to date need to be confirmed experimentally by applying (for example) a variety of methodological approaches to generate converging evidence. Furthermore, many of the studies reviewed here suffered from methodological limitations–some of which are specific to DTI methods. These DTI-related limitations (including poor-quality data, limited statistical power, the use of simplistic models of white matter organization, failure to control for cofounding variables (e.g., head motion and sociodemographic variables) and the quasi-exclusive use of FA as measure of tract integrity) have recently been comprehensively reviewed by Wang et al. (2018). Other DTI-related limitations (but which also apply to lesion-mapping functional studies of white matter tracts) include the use of a limited range of behavioral tasks and the lack of control tasks, which prevents one from probing a tract’s specific contribution to a given function.

The current review identified a certain number of subjects for further investigation. Firstly, in most of the studies reviewed here, the ILF was considered to be a single-layer white matter tract. However, the results of most recent anatomic dissection and DTI studies (e.g., Latini et al., 2017; Panesar et al., 2018) suggest that this occipitotemporal tract has at least four layers, all of which can be consistently detected in all healthy subjects. However, it is not yet clear how these four layers relate to specific cognitive, emotional or behavioral functions. For example, one might expect the fusiform branch (which connects the fusiform gyrus and the ATL) to be involved in face processing. Likewise, the cuneal branch’s cortical terminations in both the cuneal cortex and temporal-medial structures suggest that it conveys critical information for emotion processing (see **Table 1** for a tentative summary of the ILF’s layers possible functions). These hypotheses could be tested by using DTI to track each of the four layers of the ILF and by testing specific anatomic-functional correlations in both healthy and brain-damaged patients. Secondly, it was mentioned above that some functions potentially subserved by the ILF appear to be at least partially lateralized. However, to the best of our knowledge, the relationship between the ILF’s degree of anatomic lateralization and the tract’s specific functions has never been examined. This type of relationship has been demonstrated for other connective tracts and functions, such as branch II of the superior longitudinal fasciculus and spatial cognition (Thiebaut de Schotten et al., 2011). One can legitimately speculate that the right anatomic asymmetry of the ILF [as described by Latini et al. (2017)] relates to face processing. Lastly, combining fMRI, DTI, and behavioral assessments appears to be a promising avenue for disentangling the differential functional roles of the white matter tracts forming the ILF. Indeed, task-related functional activations can serve as seeds for tractography and thus help to identify specific, detailed anatomic-functional correlations between the tracked fibers and behavioral profiles. This methodology has been successfully used to highlight the involvement of basal temporal white matter tracts in face processing and prosopagnosia (Gomez et al., 2015).

**Table 1:** Summary of the functional anatomy of the ILF: a multilayered/multifunctional white matter tract.

Function	Neuropsychological syndrome	Layer^∗^	Lateralization	Pathologies involved	Main references
Face recognition	Prosopagnosia	Fusiform and lingual branches	Right	Congenital prosopagnosia	Thomas et al., 2009; Grossi et al., 2014
				Autism spectrum disorder	Koldewyn et al., 2014; Dawson et al., 2004; Boets et al., 2018
				Temporal lobe epilepsy	Drane et al., 2013; Benke et al., 2013
Object recognition	Impaired scene perception	Fusiform branch	Bilateral	Healthy volunteers	Tavor et al., 2014; Hodgetts et al., 2015
	Visual agnosia	Dorsolateral branch	Bilateral	Stroke, hematoma,	Benson et al., 1974; Meichtry et al., 2017
				Glioma awake surgery	Mandonnet et al., 2009; Coello et al., 2013
				Posterior cortical atrophy	Ortibus et al., 2012
Emotions and related processes	Hypoemotionality	Cuneal and fusiform branches	Left	Schizophrenia, 22q11.2 deletion syndrome	Ashtari et al., 2007; Tylee et al., 2017
Visual memory	Impaired visual memory	Cuneal branch	Right	Glioma	Shinoura et al., 2007
Lexical/semantic processes	Impaired semantic/lexical retrieval	Dorsolateral and fusiform branches	Left/Bilateral	Temporal lobe epilepsy	Riley et al., 2010; McDonald et al., 2014
				Glioma	Saur et al., 2008; Duffau et al., 2013; Herbet et al., 2016a,b
				Primary progressive aphasia	Mandelli et al., 2014; Tu et al., 2016
				Healthy volunteers	Ripollés et al., 2017; Hodgetts et al., 2017
Reading	Pure alexia	Dorsolateral branch	Left	Stroke, surgical injury	Epelbaum et al., 2008; Zemmoura et al., 2016
	Reading disorders	Fusiform branch	Bilateral	Prematurity, poor readers	Horowitz-Kraus et al., 2014; Arrington et al., 2017

*^∗^Deduced from cortical connections, tractographic studies, or surgical descriptions*.

In conclusion, the current review is the first to have provided general principles governing the anatomic and functional organization of the ILF. Although this knowledge is still incomplete and must be greatly extended, it may nevertheless form a solid basis for future investigations of the tract’s role in neurocognitive networks.

## Author Contributions

GH, IZ, and HD drafted, revised, and approved the final manuscript.

## Conflict of Interest Statement

The authors declare that the research was conducted in the absence of any commercial or financial relationships that could be construed as a potential conflict of interest.
